# Atomic scale simulation of carbon nanotube nucleation from hydrocarbon precursors

**DOI:** 10.1038/ncomms10306

**Published:** 2015-12-22

**Authors:** Umedjon Khalilov, Annemie Bogaerts, Erik C. Neyts

**Affiliations:** 1 PLASMANT research group, Department of Chemistry, University of Antwerp, Universiteitsplein 1, 2610 Antwerpen, Belgium

## Abstract

Atomic scale simulations of the nucleation and growth of carbon nanotubes is essential for understanding their growth mechanism. In spite of over twenty years of simulation efforts in this area, limited progress has so far been made on addressing the role of the hydrocarbon growth precursor. Here we report on atomic scale simulations of cap nucleation of single-walled carbon nanotubes from hydrocarbon precursors. The presented mechanism emphasizes the important role of hydrogen in the nucleation process, and is discussed in relation to previously presented mechanisms. In particular, the role of hydrogen in the appearance of unstable carbon structures during *in situ* experimental observations as well as the initial stage of multi-walled carbon nanotube growth is discussed. The results are in good agreement with available experimental and quantum-mechanical results, and provide a basic understanding of the incubation and nucleation stages of hydrocarbon-based CNT growth at the atomic level.

## Abstract (web summary)

Atomic scale simulation of the nucleation and growth of carbon nanotubes is essential for understanding their growth mechanism. Here, the authors look at cap nucleation of nanotubes from hydrocarbon precursors, specifically probing the role of hydrogen in the early stages of growth.

## Graphical abstract

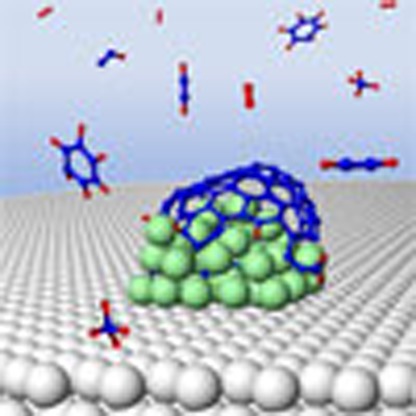

Controlling and steering the growth of carbon nanotubes (CNTs) is often believed to require control over the nucleation stage. In spite of recent advances, gaining precise control over the resulting structure proves a daunting task. Current synthesis techniques include arc discharge, laser ablation, fullerene recrystallization, catalytic chemical vapour deposition and plasma-enhanced CVD, all of which are capable of producing nanotubes with well-defined structures to a certain extent^1^,^2^,^3^,^4^. Catalytic chemical vapour deposition, in which a hydrocarbon gas is catalytically dissociated at the nanocatalyst surface at elevated temperature, is currently the most common experimental technique to grow CNTs due to its simplicity, high degree of control and scalability^5^,^6^. However, despite numerous experimental, theoretical and simulation results to understand the CNT growth, the onset (that is, incubation and nucleation stages) of CNT formation using hydrocarbon C_*n*_H_*m*_ molecules remains still unclear.

Although environmental transmission electron microscopy studies are capable of showing time-resolved details of metal-catalysed CNT nucleation and growth at the atomic level^7^,^8^, current transmission electron microscopy (TEM) resolution is still insufficient to observe atomic scale dynamics. Consequently, a variety of computational techniques including quantum mechanical, tight-binding and classical approaches have been applied to simulate CNT growth on the atomic scale^2^,^4^,^9^. A wide variety of effects, including the catalyst particle size^10^,^11^, the importance of the carbon chemical potential^12^,^13^, the necessity of a carbide phase^14^,^15^,^16^,^17^, the importance of metal-mediated defect healing^18^,^19^, the interaction between metal and carbon network^20^,^21^, the influence of growth precursor, using either C atoms or C_2_ dimers^22^ or energetic C-ions^23^ as input gas, as well as the influence of ion bombardment^23^,^24^ and the effect of applying an electric field^25^ have been computationally studied^2^,^3^,^4^,^9^,^26^.

Until now, however, none of these studies considered cap nucleation from a hydrocarbon growth precursor. Instead, pure carbon (either as C or C_2_) is invariably used as carbon source. Hence, instantaneous hydrocarbon decomposition is implicitly assumed, whereas the role of hydrogen is never considered^3^. Only very recently, the first dynamic simulation studies appeared on related processes using hydrocarbon molecules as carbon feedstock^4^. Somers *et al.* and Liu *et al.* employed reactive molecular dynamics (MD) simulations to study the interaction of methane and methane-derived radicals with Ni-surfaces in the context of plasma catalysis^27^,^28^,^29^,^30^,^31^. Shibuta *et al.* performed *ab initio* MD simulations to study the decomposition of CH_4_ and C_2_H_4_ molecules on a Ni(111) surface^32^,^33^ and the reactivity of CH_4_ on a Cu(111) surface^34^ in the context of metal-catalysed graphene growth. Recently, Wang *et al.*^35^ studied the interaction of acetylene with a Fe_38_ cluster by density-functional tight-binding simulations and concluded that cap formation is not a necessary condition for single-walled CNT (SWNT) nucleation and large hydrocarbon clusters can grow before hydrogen abstraction would allow the formation of a carbon-only tubular structure. In contrast, however, experimental *in situ* studies by Hofmann *et al.*^7^ and Yoshida *et al.*^8^ demonstrated cap lift-off preceding SWNT growth on a surface-bound Fe or Ni nanocatalyst using a C_2_H_2_ feedstock. In our previous work, we also demonstrated the formation of such vertical graphene nanowalls, which transform to a horizontally oriented carbon network, partially covering the catalyst surface^36^. So far, however, there have been no reports on cap and nanotube nucleation from hydrocarbon species.

In this work, we report on CNT cap nucleation using different hydrocarbon species at different temperatures to unravel the initiation of CNT growth by combined reactive MD and time-stamped force-bias Monte Carlo (tfMC) simulations^37^. We find that the competition between (re)hydrogenation and dehydrogenation processes during the incubation stage is critical for the dynamics of the cap formation and subsequent CNT growth process.

## Results

### Dehydrogenation degree

It is known that hydrogen may either enhance CNT growth^38^ or etch the growing CNT^39^, and thus has a dual role. Both effects depend on the H concentration at the surface. Therefore, steering the H concentration during the CNT growth is very important^38^,^39^,^40^,^41^. Until now, however, the precise role of adsorbed H atoms in the onset of CNT growth has not yet been studied down to the atomic level^3^,^4^. As demonstrated below, the growth from hydrocarbon gas precursors depends on the adsorption rate of C_*n*_H_*m*_ precursors, and the desorption rate of H_2_ molecules and C_*n−x*_H_*m−y*_ species. The calculation of adsorption and desorption rates is thus elementary to assess the dehydrogenation degree of the adsorbed precursor and the catalyst cluster during the growth. The *k*-coefficient is introduced to estimate the dehydrogenation degree^36^, to analyse the CNT growth stages:


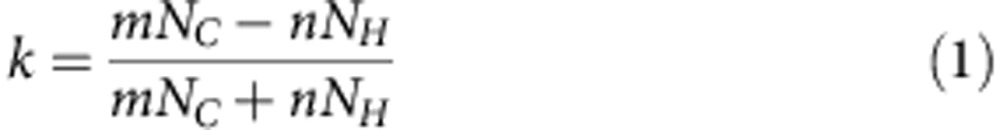


where *n* and *m* are the number of carbon and hydrogen atoms, respectively, in the C_*n*_H_*m*_ molecule and *N*_*C*_ and *N*_*H*_ are the total number of adsorbed carbon and hydrogen atoms in the cluster, respectively. The *k*-coefficient (*k*∈[0,1]) allows to easily differentiate between physisorption of the hydrocarbon molecule on the catalyst surface (*k=0*) and complete dehydrogenation of the adsorbed molecule and the catalyst (*k=1*). Note that the coefficient depends on both the H_2_ desorption rate and the H-arrival rate, but it does not depend on how the precursor decomposes.

**Figure 1:**
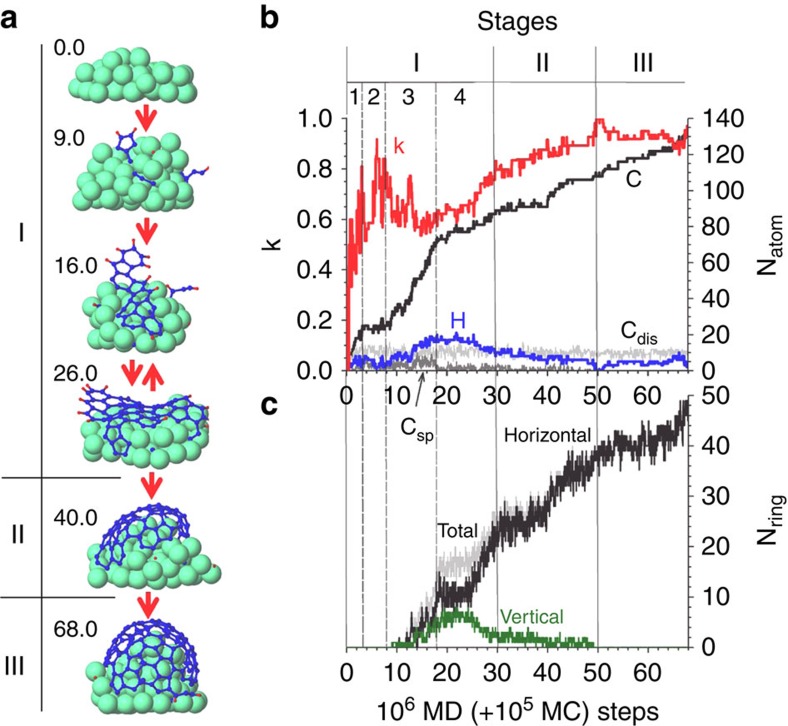
Analysis of the CNT growth stages. (**a**) Evolution of the CNT cap nucleation process up to 6.8 × 10^7^ (+2 × 10^6^ MC) MD-steps; Ni, C and H atoms are coloured light green, blue and red, respectively; (**b**) evolution of the *k*-coefficient and the number of C atoms (including dissolved C_dis_ and sp-hybridized C_sp_ carbon atoms) and H adatoms on and in the catalyst nanocluster as a function of the simulation time; (**c**) evolution of vertical (free-standing), horizontal (adsorbed on the nanocluster) and total number of carbon rings as a function of the simulation time.

It is well known that different structures may be formed, depending on the adsorption (hydrogenation) and desorption (dehydrogenation) rates, temperature, partial pressure of the hydrocarbon molecule and the H-content in the molecule^1^,^40^,^42^. Thus, methane (CH_4_), acetylene (C_2_H_2_) and benzene (C_6_H_6_) will typically yield different structures. Yet, we find that the overall chemical nature of the hydrogenation and dehydrogenation processes is very similar for these molecules, and we can therefore generalize all cases in a single mechanism, as depicted in Fig. 1.

The figure shows the evolution of the cap formation, the number of C and H atoms on and in the nanocluster, the formation of carbon hexagonal, pentagonal and heptagonal rings, as well as the evolution of the *k*-coefficient as a function of the simulation time. Based on this evolution, we distinguish three main stages in the onset of CNT growth: incubation (stage I), cap formation (stage II) and continued growth (stage III).

### Stage I

The incubation stage is vital in understanding the onset of CNT growth. Although this stage has been studied before by many researchers, there is currently little knowledge on its role in relation to the subsequent cap formation stage, and in particular in the case of hydrocarbon-based growth (see Supplementary Table 1 for a comparison in this regard between earlier work and the current work). We therefore here first analyse stage I, which we divide in four substages.

In the first substage (or supersaturation stage), all C–H and C–C bonds of the impinging hydrocarbon molecule are gradually broken and the C atoms eventually dissolve into the pure Ni_55_ nanocluster (Supplementary Fig. 1) in agreement with earlier studies^32^,^35^,^36^. H adatoms freely diffuse over the nanocluster and recombine with other H atoms on the surface to desorb as H_2_ from the nanoparticle^28^,^36^. Also, at high-impingement flux and low temperature, some adsorbed molecules (for example, C_6_H_6_ or CH_4_) do not completely dehydrogenate^36^,^43^. The number of adsorbed/dissolved carbon atoms quickly increases due to the fast hydrogen desorption and therefore the *k*-coefficient quickly rises towards one (Fig. 1b, substage 1, see *k*). Generally, in this substage, this ‘C_*n*_H_*m*_ adsorption/dissociation and H_2_ desorption' scenario continues until the cluster is (super)saturated^13^. Owing to the dissolution of carbon in the particle, the wetting angle of the Ni_*x*_C_*y*_ drop increases (cf. Fig. 1a, time 0.0 and time 9.0), in agreement with both experiment^44^ and simulation results^45^.

The second substage of stage I begins when initial carbon structures start to appear on the catalyst surface. After the supersaturation, the dissociation rate of newly adsorbing hydrocarbon molecules slows down considerably (Fig. 1b, substage 2). As a result, the adsorption rate of hydrocarbon molecules strongly decelerates. Although C_*n*_H_*m*_ adsorption is now close to zero, H_2_ desorption still occurs and thus the *k*-coefficient continues to increase (Fig. 1b, see *k*). Simultaneously, dissolved carbon atoms start to segregate at the surface, allowing new appearing C atoms (by dissociation of the few adsorbing C_*n*_H_*m*_ molecules) to dissolve, in accordance with the vapour–liquid–solid mechanism^46^,^47^. The number of dissolved carbon atoms thus remains roughly constant after the supersaturation stage, amounting to ∼15 at% (Fig. 1b, see C_dis_). Segregated C atoms diffuse over and through the catalyst (sub)surface until recombining with other carbon atom(s) to form initial surface carbon structures, *viz.* C_2_ dimers, sp^2^ and sp^3^ C atoms and short C_y_ polyyne chains without (*y≤5*) or with one or two H-terminations (*y≤4*; Fig. 1a, time 9.0; Fig. 1b, see C_sp_). Note that due to the Gibbs–Thompson effect^48^,^49^, the Ni_55_ cluster is liquefied and consequently special crystallographic planes disappear after thermalization and thus no segregation along specific crystallographic planes is observed during the simulation.

The onset of the third substage (or formation of vertical nanowalls) of stage I is characterized by the formation of the first carbon ring on the surface. We find that the initial pentagon^50^ (Fig. 1a, 9.0 and Fig. 1c) or hexagon ring^51^ can be formed in four distinct ways: (i) by incorporation of segregated or surface C atoms in an existing surface carbon structure; (ii) by folding of a polyyne (C_*y*_, C_*y*_H or C_*y*_H_2_) chain into a ring structure (Supplementary Fig. 2); (iii) in the case of acetylene: by direct interaction of gas-phase C_2_H_2_ with surface C-structures such as a carbon trimer thereby forming a pentagon; or (iv) in the case of benzene: by incorporation of the impinging molecule in an existing surface carbon structure after partial dehydrogenation (see Supplementary Fig. 1, C_6_H_6_ case). Although the former two processes have been described earlier in terms of the cross-linking mechanism proposed by Eres *et al.*^35^,^52^ and the ‘pentagon-first' mechanism presented by the group of Irle and Morokuma^53^, the latter two processes have not yet been described before, as they are specific to hydrogen-containing growth precursors.

The appearing pentagons and hexagons are mobile and may either concatenate or connect with (sub)surface carbon atoms or other carbon structures to form a small graphitic nucleus. Meanwhile, diffusing H adatoms interact with and add to the various carbon surface structures including partially dissociated C_*n*_H_*m−x*_ hydrocarbons, polyyne chains and incipient pentagon–hexagon networks on the surface (‘rehydrogenation'), or they may recombine with other H atoms to desorb as H_2_ from the surface (‘dehydrogenation')^36^. Owing to the presence of the carbon surface structures, the H atoms cannot diffuse freely over the surface. As a result, the H_2_ desorption rate decreases, and the number of surface H atoms significantly increases. This leads to a decrease of the *k*-coefficient (Fig. 1b, see H and *k*, substage 3). When rehydrogenation occurs, the surface carbon structures including polyyne chains and graphene-like networks become partially hydrogenated and vertically free-standing graphene sheets or carbon nanowalls are formed on the catalyst surface (Supplementary Fig. 3), corresponding to recent *ab initio* MD^33^ and density-functional tight-binding-based MD studies^35^. Subsequently, these parallel graphene-like sheets may transform to hydrogen-terminated few-layer graphene and thereby form the initial structures leading to lift-off of a multi-walled CNT (MWNT) or a carbon nanofiber as proposed by Hofmann *et al.*^7^ Our results indicate that while a high temperature and low H-content yield the formation of SWNTs, a low temperature and high H-content favour MWNT nucleation (Supplementary Fig. 4), in qualitative agreement with the general experience of CVD experiments, that is, low-temperature CVD (600–900 °C) yields MWNTs, whereas high-temperature (900–1200 °C) conditions favour SWNT growth^42^.

Also, we observe that smaller free-standing graphene patches as well as other initial carbon structures can be etched if the flux of H atoms is sufficiently high, as is the case for CH_4_ as growth precursor in our simulations, which is in agreement with experimental reports using methane as growth precursor above 800 °C (ref. 40). This indicates that the growth rate of CNTs from CH_4_ can be much lower compared with growth from C_2_H_2_ and C_6_H_6_ due to the higher (re)hydrogenation rate compared with the dehydrogenation rate.

Thus, in this substage, both horizontally and vertically oriented carbon patches can exist (Fig. 1a, time 16.0 and time 26.0), due to competition between dehydrogenation and rehydrogenation, which in turn strongly depends on the growth temperature, type of hydrocarbon species and their gas-phase pressure (or flux) as well as the catalyst-substrate interaction^36^. The competition between rehydrogenation, dehydrogenation and H etching thus explains how unstable carbon structures may appear in the incubation stage, as for example, observed during *in situ* TEM observations^8^. Such studies indeed found protrusions of various unstable carbon structures before eventual cap appearance and lift-off.

In the fourth and final substage of stage I, vertical nanowalls and horizontal nanosheets can be simultaneously found on the catalyst surface. However, the vertical free-standing networks invariably and irreversibly transform to horizontal networks covering the surface. Consequently, the graphitic network starts to gradually cover the nanocluster surface.

**Figure 2:**
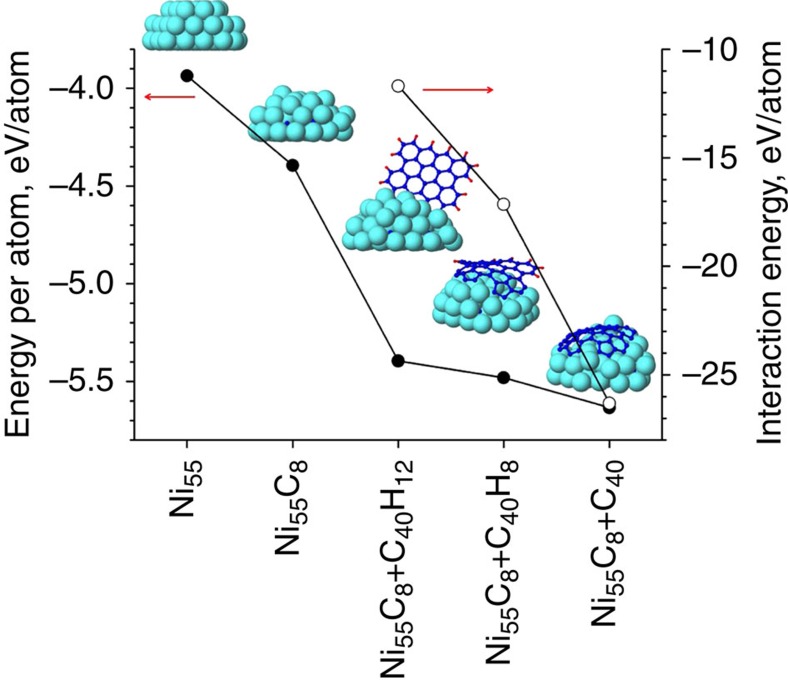
Energy of vertical nanowall versus horizontal nanosheet. Potential energies per atom (solid circles) of the pure Ni_55_ nanocluster, Ni_55_C_8_ nickel carbide cluster, totally (C_40_H_12_) and partially (C_40_H_8_) H-terminated graphene walls as well as a C_40_ graphene sheet on the Ni_55_C_8_ nickel carbide; interaction energies (open circles) between the C_40_H_12_ or C_40_H_8_ wall and the Ni_55_C_8_ nanocluster, as well as between the C_40_ graphene sheet and the Ni_55_C_8_ nanocluster.

In Fig. 2, potential energies per atom of five selected samples, which are found during the aforementioned stages, are shown (solid circles). From the figure, it is clear that horizontal graphene patches, covering the surface, are energetically favoured over their vertical free-standing counterparts. Accordingly, the interaction energy between a C_40_ graphene patch and the nanocluster significantly increases when the vertically oriented carbon nanowall (Ni_55_C_8_+C_40_H_12_ and Ni_55_C_8_+C_40_H_8_) transforms to the horizontally oriented nanosheet (Ni_55_C_8_+C_40_), after elimination of their edge-terminated H atoms (open circles in Fig. 2).

As a result of this transformation, the rate of hydrocarbon adsorption decreases due to the decreasing free-metal surface area. The increase in number of carbon atoms therefore drops relative to the previous stage, and remains constant throughout the rest of the simulation time (Fig. 1b, see C, substage 4). Likewise, the number of H adatoms decreases due to both a small adsorption rate of C_*n*_H_*m*_ and the high H_2_ desorption rate (Fig. 1b, see H) and thus the *k*-coefficient rises again. When H atoms connect to the edges of the horizontal graphene patches, the carbon sheets can only partially stand upright on the cluster (Fig. 1a, time 26.0), limiting the number of free-standing rings in this stage (Fig. 1c). Also, the number of polyyne chains with or without H-termination (that is, C_*y*_ or C_*y*_H) becomes negligible for the rest of the simulation.

### Stage II

This second main stage is characterized by the appearance of a carbon cap on the cluster, which is essential for the nucleation of a CNT (refs 7, 8). In this stage, the adsorption rate of C atoms does not change, whereas the density of H atoms continues to decrease. The *k*-coefficient can therefore rise close to one (Fig. 1b, see *k*, stage II). The dehydrogenation rate is now somewhat slower compared with fourth substage of stage I. Also, the horizontally oriented graphene-like patches start to lose their H atoms from their edges. In agreement with first-principles calculations^50^, the cap on a curved Ni-cluster is more stable than a graphene sheet (Fig. 2), and at a sufficiently high temperature a cap hence develops and eventually lifts off (Fig. 1a, time 40.0).

**Figure 3:**
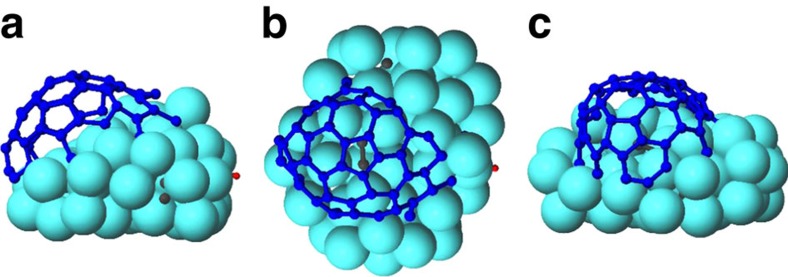
A carbon cap. Side view (**a**), top view (**b**) and front view (**c**) of a carbon cap formed under low-flux C_2_H_2_ impacts. Surface and subsurface C and C_2_ species are coloured grey for the sake of clarity.

In Fig. 3, a cap as formed during low-flux C_2_H_2_ impacts at high temperature is shown. In the cap, the central pentagon ring is surrounded by five hexagon rings (Fig. 3b) indicating essentially defect-free CNT nucleation. The formation of this carbon dome, containing a hexagon-surrounded pentagon ring or a hemispherical fullerene structure, corresponds very well with both *in situ* experiments^7^,^54^ and *ab initio* MD simulations^55^.

After cap formation, a metal step appears between the tube and the substrate due to the smaller diameter of the cap than the diameter of the Ni cluster (Fig. 3). Although the formation of such steps is important for the growth of MWNTs (refs 7, 54, 56), we observe that the growing nanotube actually forces its shape onto the Ni cluster (Fig. 1a, time 68.0) and the metal steps consequently disappear during the growth, corresponding to observations in *in situ* experiments for SWNT growth^7^.

Our simulations demonstrate that cap nucleation in CNT growth from hydrocarbons does not require the formation of long polyyne chains. This is in contrast to the many simulations predicting the formation of such chains on the surface when pure, hydrogen-free carbon is used as growth precursor^4^. Although the presence of such chains during the nucleation and growth has been suggested before^52^, no direct experimental evidence for their occurrence, however, has been produced yet. Indeed, our simulations demonstrate that cap nucleation does not require the formation of such long polyyne chains.

At low temperature, we do not observe the formation of a cap, corresponding to earlier MD simulations demonstrating how lift-off requires a weak work of adhesion, high growth temperature (or sufficient thermal kinetic energy) and/or a large catalyst or a low curvature of the catalyst surface^20^,^21^. Finally, note that in our simulations the substrate-bound semi-spherical nanocatalyst is not entirely encapsulated by the carbon network at low temperature and a significant part of the network edge is quickly saturated by H atoms.

### Stage III

In the final stage, the CNT continues to grow on the substrate-bound catalyst surface (Fig. 1a, time 68.0) due to the incorporation of the segregated C atoms, diffusing C adatoms or through direct adsorption of partially dehydrogenated C_*n−x*_H_*m−y*_ fragments near the metal/cap interface followed by dehydrogenation.

**Figure 4:**
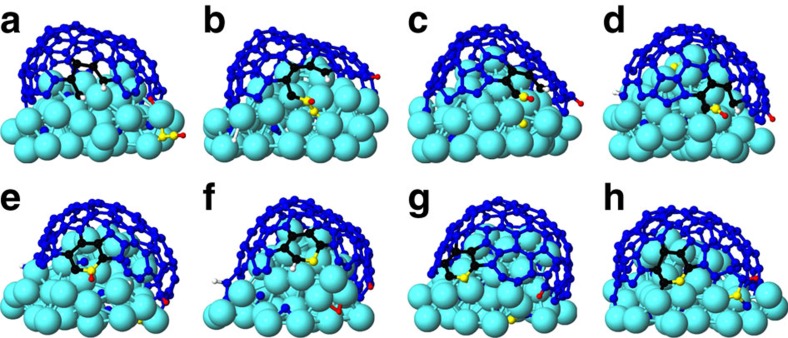
C diffusion/incorporation mechanism. C diffusion and its incorporation through several steps (**a**–**h**) after adsorption and dissociation of C_2_H_2_. C and H atoms of the impinging molecule are coloured yellow and red, respectively. C atoms in newly formed hexagon rings at the nanotube edge are coloured black.

In Fig. 4, C diffusion and its incorporation mechanism during CNT growth is shown for the case of C_2_H_2_ impacts. When the molecule impinges on the cluster, it quickly loses one of its H atoms (Fig. 4a). Consequently, the remaining ethynyl (C_2_H) radical diffuses over the Ni surface before it incorporates into the nanotube base (Fig. 4b). After incorporation, one of the C atoms of the C_2_H radical diffuses into the cluster (Fig. 4c) while the H atom diffuses over the surface (Fig. 4d). The C atom from the remaining part of the ethynyl radical binds to a C atom in the tube edge and eventually a new hexagon ring appears at the tube-cluster interface (Fig. 4e). As a result, the second H atom is also eliminated from the ring and it can subsequently also diffuse over the surface (Fig. 4f). Finally, a C atom from the bulk may segregate at the surface (Fig. 4g) and subsequently incorporate into the CNT edge (Fig. 4h).

Our results indicate that C atoms initially diffuse into the catalyst cluster after C_*n*_H_*m*_ decomposition (Supplementary Fig. 1), according to the vapour–liquid–solid model^46^ and in agreement with various experimental observations^8^,^54^,^57^, two scenarios turn out to be possible after supersaturation: (a) either C atoms continue to diffuse into the cluster, provided they completely lose their hydrogens, or (b) they diffuse over the catalyst surface, in case they keep or only partially lose their hydrogen. In either case, the C atoms eventually incorporate into the growing network (Supplementary Figs 5–8).

Finally, note that in the case of high H-content (for example, CH_4_ and C_6_H_6_) at high flux conditions, the nanotube either grows very slowly or grows with many defects due to the presence of a significant number of H atoms^41^. These H atoms can react with defective sites of the CNT when hydrocarbon molecules dissociate on the nanocluster surface. In the last stage, the *k*-coefficient remains very close to one, indicating the presence of some H adatoms, signalling a suitable growth condition when using hydrocarbon feedstock (Fig. 1b, see H, stage III).

## Discussion

The overall results demonstrate the important role of the dehydrogenation degree in determining the various growth stages. In the first two substages of the incubation stage (stage I), adsorbed C_*n*_H_*m*_ species quickly decompose and lose their H atoms. Consequently, C atoms dissolve into the cluster (Supplementary Fig. 1). In all hydrocarbon impact cases, the *k*-coefficient rises towards its maximum (that is, *k* tends to 1), as the H_2_ desorption rate is much higher than the H-arrival rate. Owing to the high degree of dehydrogenation, the system quickly saturates with C atoms and consequently initial C-structures appear on the cluster. Also in substage 4 (formation of horizontal graphene sheets on the surface) of stage I and in stage II (formation of a carbon cap), *k* is found to increase. When *k*≈1, the effect of hydrogen on the nucleation and growth process is negligible, and these stages are therefore very similar to the growth stages as found in growth simulations based on C-only precursors.

In the third substage of the incubation stage, *k* decreases significantly (that is, *k* tends to 0): the increase in the number of adsorbed H atoms exceeds the number of desorbed H_2_ molecules per unit of time: after supersaturation, surface C atoms increase fast and diffusing H atoms quickly connect to initial C-structures (rehydrogenation) rather than to find other H atoms to desorb from the surface as H_2_ (dehydrogenation). Such fast hydrogenation eventually leads to the formation of the free-standing graphitic patches (see Supplementary Figure 3). Therefore, a low dehydrogenation degree is characterized by the appearance of H-terminated free-standing graphene patches in the system. In this stage, the effect of the presence of hydrogen cannot be ignored, and simulations based on pure C-precursors cannot provide any information on this growth stage.

In the third stage of the nucleation process, the carbon cap covers a significant part of the catalyst nanoparticle and only a small area of the catalyst surface remains available for hydrocarbon species to impinge and dissociate before H_2_ desorption may occur (Supplementary Figs 6–8). The combination of a low growth rate, a high concentration of C atoms in the system, a low-sticking probability of gas-phase species and the H desorption and H arrival rates being equal to each other leads to the *k-*coefficient to be continuously high and close to one (that is, constant *k*_*max*_), which is the essential condition for continued CNT growth from hydrocarbons.

In summary, using combined MD/Monte Carlo simulations, the nucleation of SWNT caps from hydrocarbon precursors (CH_4_, C_2_H_2_ and C_6_H_6_) is studied for the first time. The complete nucleation mechanism consists of three main consecutive stages: incubation (consisting of four substages), cap formation and continued growth.

In the incubation stage, impinging hydrocarbon molecules may contribute to initial ring formation after supersaturation, in addition to earlier suggested mechanisms. Free-standing graphene nanowalls as well as a horizontal carbon network are found on the catalyst cluster due to competition of (re)hydrogenation and dehydrogenation processes. We also find that dehydrogenation, rehydrogenation and H etching can explain a number of experimental observations: (1) the appearance of unstable carbon protrusions during the incubation stage in *in situ* transmission electron microscopy-observations; (2) the onset of MWNT nucleation through the formation of a multi-layer graphene structure in the case of low temperature and high H flux; and (3) the lower growth rate of CNTs from CH_4_ compared with growth from C_2_H_2_ and C_6_H_6_.

In the second stage, the cap formation mechanism proceeds from a horizontal carbon nanosheet through H-elimination. We find that cap nucleation in CNT growth from hydrocarbons does not require the formation of long polyyne chains. At low temperature, the substrate-bound semi-spherical nanocatalyst is not entirely encapsulated by the carbon network and a significant part of the network edge is quickly saturated by H atoms.

In the growth stage, C atoms may either diffuse into the cluster after full dehydrogenation, or diffuse over the surface in the case of partial dehydrogenation. In either case, they are found to eventually incorporate into the carbon network. During the growth, the growing nanotube is observed to force its shape onto the Ni cluster and consequently metal steps gradually disappear.

We thus conclude that the dehydrogenation degree can distinguish various growth (sub) stages and thus control over the *k-*coefficient is a highly important factor in controlling CNT nucleation and growth.

## Methods

### Hybrid MD/tfMC technique and ReaxFF

Combined reactive MD and tfMC simulations^37^,^58^,^59^ are used to simulate the catalysed CNT growth process. In tfMC calculations, all the atoms in the system are displaced at once in every time step with unit probability, and thus generate a system evolution in a MD-like fashion, instead of, for example, single-particle moves common in Metropolis MC (ref. 37). This technique can therefore very efficiently be coupled to canonical MD simulations: the MD module accounts for the impacts of the growth species on the catalyst surface and for the initial chemical reactions taking place during the first few picoseconds, whereas the tfMC module takes care of the longer timescale relaxation of the system^37^. In a single tfMC simulation step, each atom is displaced in a direction that is dependent on the force (deterministic component), as well as on the temperature (stochastic component)^59^. The typical step size in tfMC is about 0.1 Å, which is at least one order of magnitude longer than in MD (∼0.01 Å)^59^,^60^. Bond dissociation and other activated processes occur in MD only due to thermal fluctuations, rendering these events to be rare. In tfMC, in contrast, the random component in the displacement algorithm allows atoms to move against the force, and thus allow the system to cross transition states and attain a more stable configuration much more quickly than is possible in MD. Although it is clear that the exact system dynamics are not reproduced by tfMC, it has been demonstrated that tfMC correctly accounts for detailed balance, provides a realistic (albeit not exact) dynamical path and reproduces end configurations in full agreement with (very long) MD simulations. Both during the MD and the tfMC cycles, ReaxFF (refs 61, 62) is applied to properly describe the bond dissociation and formation processes, that is, the C–C bond dissociation, dehydrogenation, rehydrogenation and H_2_ formation as well as other reactions. Currently, accelerated MD simulations are carried out to further extend the time scale of CNT growth simulations^63^.

### Simulation details

A Ni_55_ nanocatalyst is equilibrated at the desired growth temperature using the canonical Bussi thermostat^64^. The nanocatalyst is physisorbed on a virtual Al or Si substrate, employing a *z*-integrated Lennard-Jones potential^36^. Subsequently, methane (CH_4_), acetylene (C_2_H_2_) or benzene (C_6_H_6_) molecules are allowed to impinge on the cluster. Throughout the simulation, the total number of gas-phase hydrocarbon species is kept constant. When a hydrocarbon molecule adsorbs on the Ni cluster, the resulting structure is allowed to relax by application of tfMC (refs 37, 60). During the relaxation, no new molecules are allowed to impinge on the cluster. Application of the combined tfMC/MD technique was previously demonstrated to allow relaxation processes in general and the simulation of the CNT nucleation process in particular much more efficiently compared to pure MD simulations^37^,^58^. All simulations are performed at a temperature in the range of 1000–2,000 K controlled by the canonical thermostat^64^.

## Additional information

**How to cite this article**: Khalilov, U. *et al.* Atomic scale simulation of carbon nanotube nucleation from hydrocarbon precursors. *Nat. Commun.* 6:10306 doi: 10.1038/ncomms10306 (2015).

## Supplementary Material

Supplementary Information Supplementary Figures 1-8 and Supplementary Table 1
