# Supplementary material for: Atomic scale simulation of carbon nanotube nucleation from hydrocarbon precursors
Source: Nat Commun. 2015 Dec 22;6:10306. doi: 10.1038/ncomms10306 (PMC4703880; doi:10.1038/ncomms10306)
Supplement: Supplementary Information — Supplementary Figures 1-8 and Supplementary Table 1 [file ncomms10306-s1.pdf]

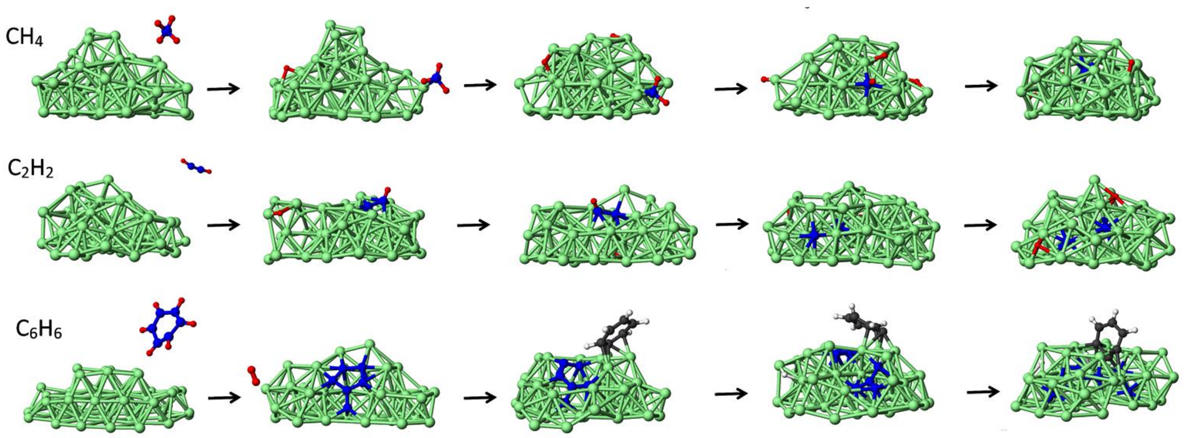

Supplementary Figure 1 | Bulk-diffusion of C atoms. C diffusion after dissociation of an adsorbed C_nH_m (methane CH_4, acetylene C_2H_2 or benzene C_6H_6) molecule.

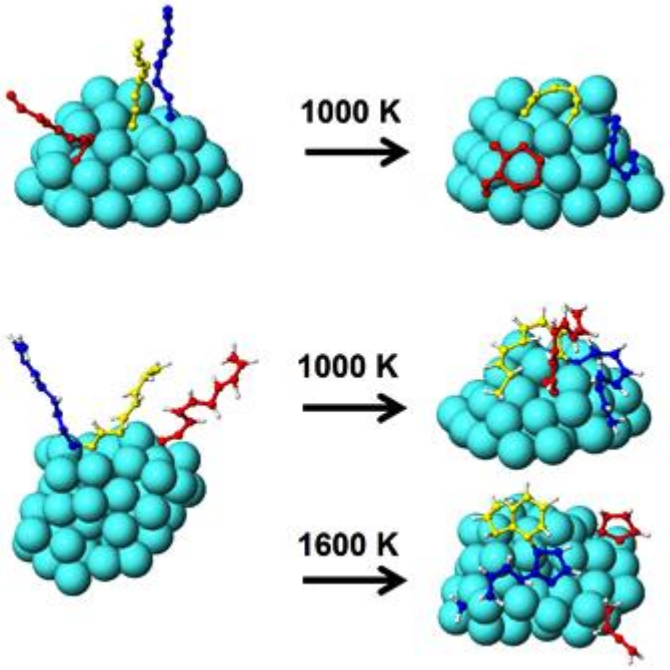

Supplementary Figure 2 | Polyyne chains. Folding of hydrogenated and pure-carbon polyyne (-like) chains into a ring structure at 1000 K and 1600 K.

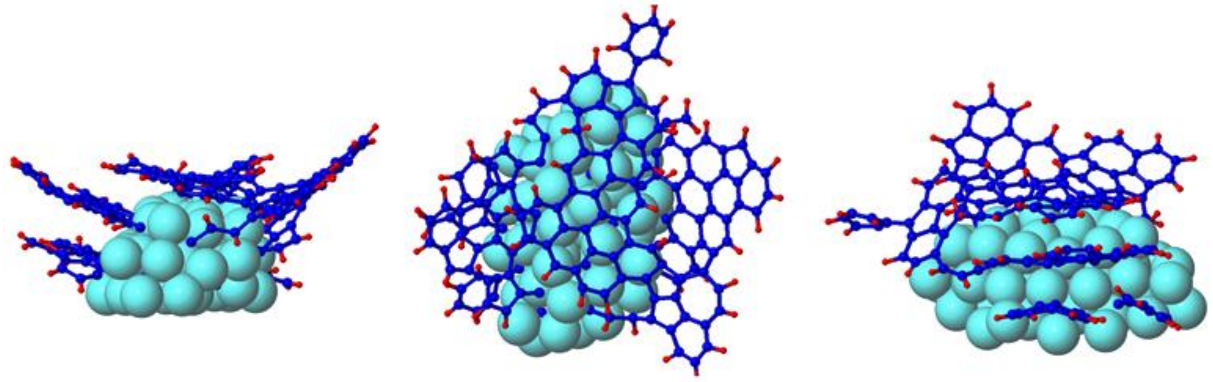

Supplementary Figure 3 | Parallel carbon nanowalls. Formation of vertically-oriented graphene patches from side view (left), top view (middle) and front view (right). The figure shows that the patches almost completely consist of hexagons, and the edges are fully hydrogenated.

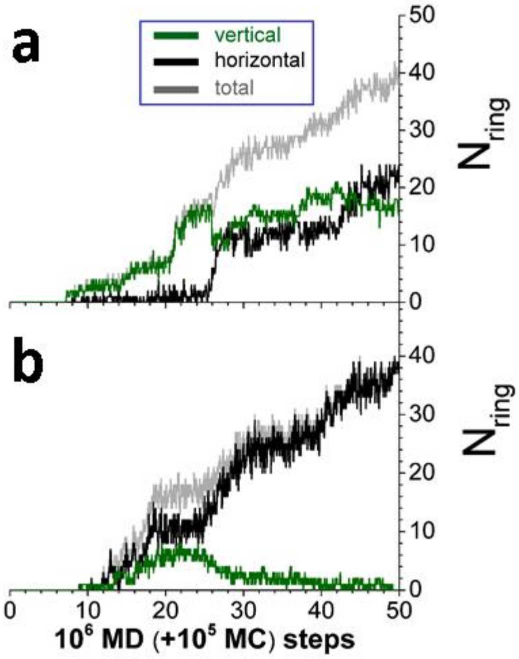

Supplementary Figure 4 | Temperature effect. Evolution of vertical (free-standing), horizontal (adsorbed on the nanocluster) and total number of carbon rings as a function of the simulation time for C_6H_6 at 1600 K (**a**) and C_2H_2 at 2000 K (**b**).

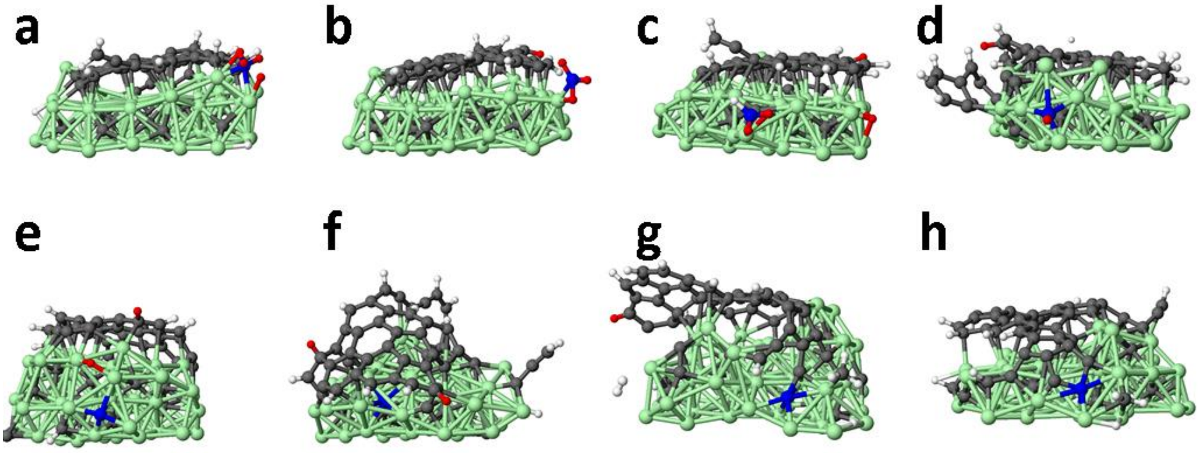

Supplementary Figure 5 | C diffusion/incorporation in stage I.4. C diffusion and incorporation (**a**-**h**) in the case of CH_4 impact.

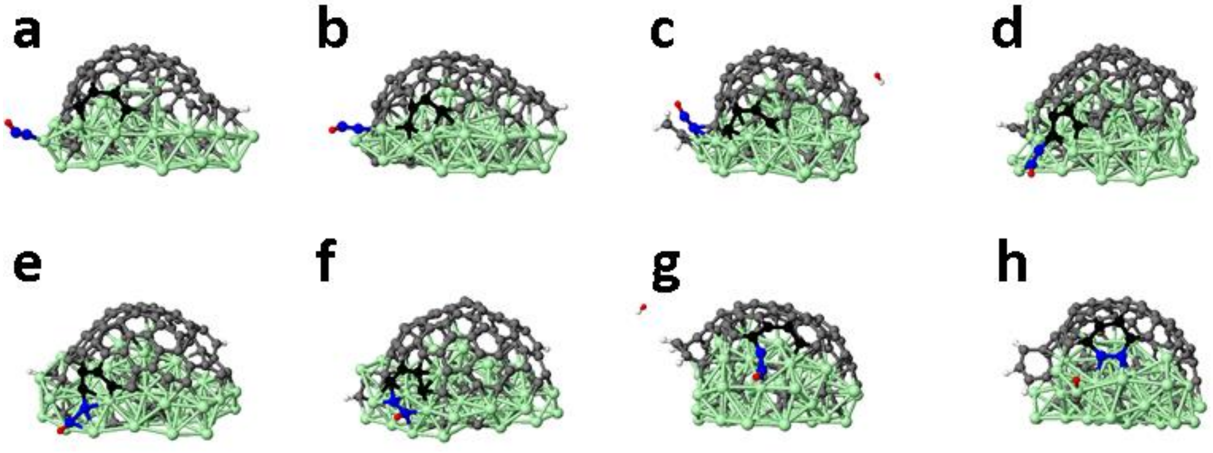

Supplementary Figure 6 | C diffusion/incorporation in stage II. C diffusion and incorporation (**a**-**h**) in the case of C_2H_2 impact.

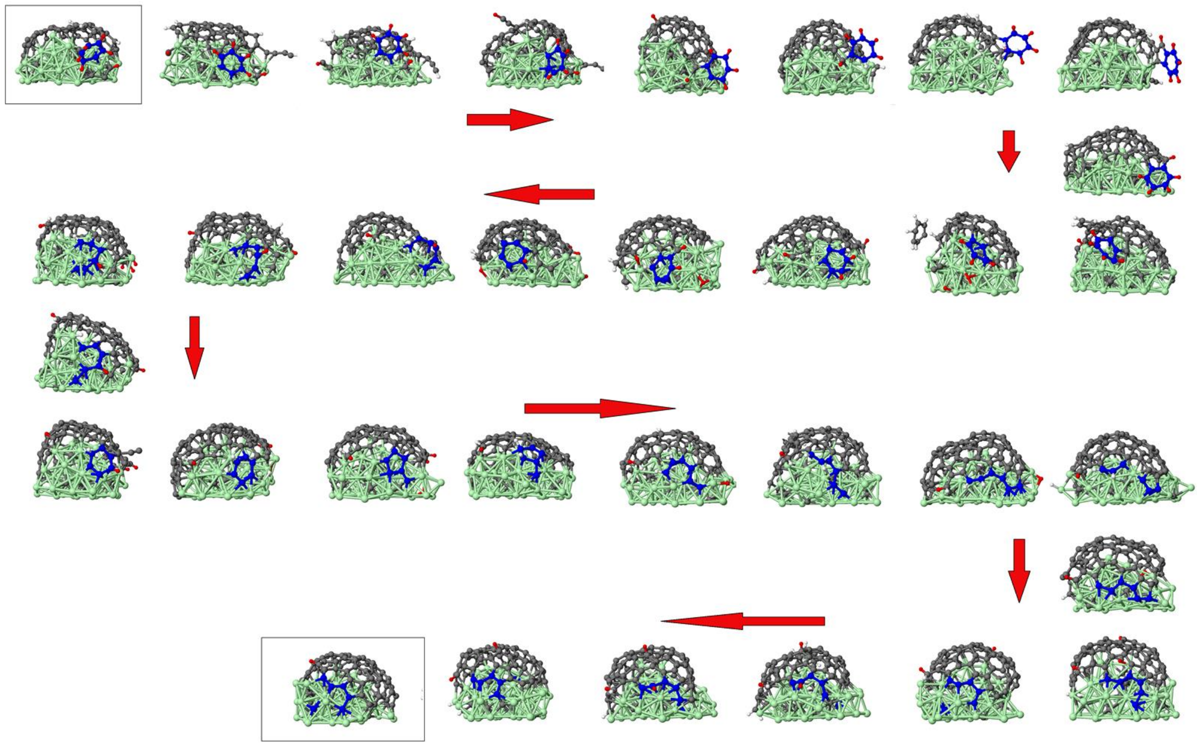

Supplementary Figure 7 | C diffusion/incorporation in stages II-III. C diffusion and incorporation in the case of C_6H_6 impact.

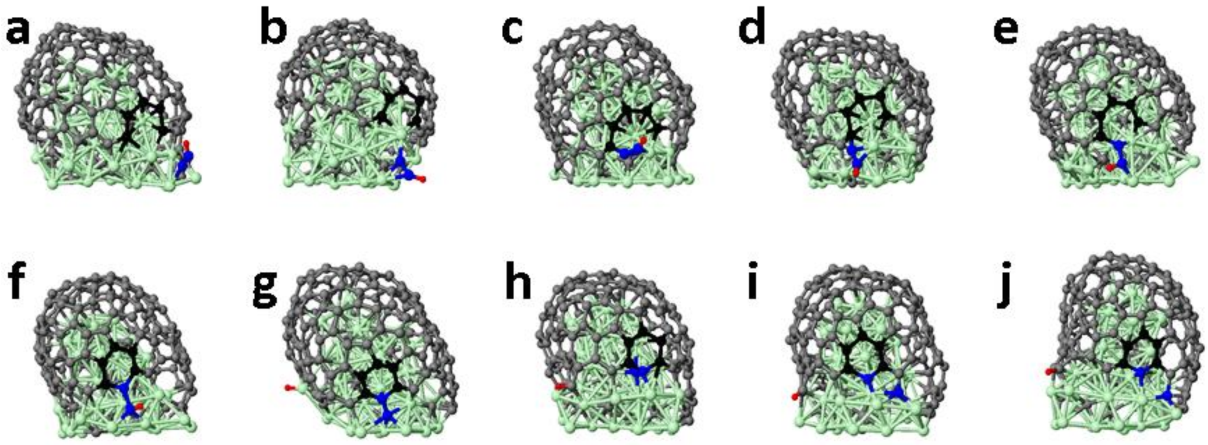

Supplementary Figure 8 | C diffusion/incorporation in stage III. C diffusion and incorporation (**a**-**j**) in the case of C_2H_2 impact.

Supplementary Table 1 | Comparison with related results from literature. Comparison of the current study with existing simulation and experimental studies.

Reference	Method		Most relevant results	Corresponding growth (sub-) stages in this work
Y. Shibuta et al. *Chem. Phys. Lett.* **565,** 92-97 (2013)	**modeling**	ab initio MD simulations	Low adsorption probability of methane (CH_4) molecule on Ni(111) surface.	Stage I (substage 1)
R. Arifin et al. *Phys. Chem. C* **119,** 3210-3216 (2015)		ab initio MD simulations	After ethylene dissociation and dehydrogenation, C_2 dimers remain on Ni(111) surface, while C atoms diffuse into the crystal.	Stage I (substage 2)
			Formation of standing $C{=}CH_3$ fragments on Ni(111) surface after impinging C_2H_4 molecule due to rehydrogenation.	Stage I (substages 1 and 2)
Y. Wang et al. *Carbon* **72,** 22-37 (2014)		Non-equilibrium quantum chemical MD simulations	Easy adsorption of acetylene (C_2H_2) on Fe_{38} cluster and consequent formation of ethynyl (C_2H) radical.	Stage I (substage 1)
			Formation of standing graphene-like nanowalls on the metal cluster.	Stage I (substage 3)
H. Yoshida et al. *Nano Lett.* **8,** 2082-2086 (2008)	**experiment**	in situ ETEM observations	C diffusion into Fe nanocluster.	Stage I (substage 1)
			Prior to SWNT nucleation, various unstable carbon cages protrude and disappear.	Stage I (substages 3 and 4)
Hofmann et al. *Nano Lett.* **7,** 602-608 (2007)		ETEM and in situ time-resolved XPS observations	A carbon cap first emerges with a diameter smaller than the catalyst cluster. The carbon network expands by lifting off from the catalyst particle, which thereby restructures. The growing nanotube forces its shape onto the Ni cluster.	Stages II and III
